# Effects of dam metabolic profile and seasonality (Spring vs. Winter) on their offspring’ metabolism, health, and immunity: maternal factors in dairy calves’ analytes

**DOI:** 10.3389/fvets.2024.1424960

**Published:** 2024-07-11

**Authors:** Fernanda Carolina Ramos dos Santos, Bianca Paola Santarosa, Felipe Eduardo Dal Más, Karen Nascimento da Silva, Érica Cristina Bueno do Prado Guirro, Viviani Gomes

**Affiliations:** ^1^Department of Internal Medicine, School of Veterinary Medicine and Animal Science, University of São Paulo, São Paulo, Brazil; ^2^Departament of Veterinary Science, Federal University of Paraná, Palotina, Paraná State, Brazil

**Keywords:** calves, dairy cattle, inflammation, innate immune response, maternal factor, season, transition period in dairy cows

## Abstract

Maternal status during the transition period can significantly impact the health and performance of Holstein dairy calves, with lasting effects on various variables. However, the relationship between maternal late gestation metabolic status, seasonality, and their impact on offspring remains unclear. This study aimed to assess the influence of maternal variables at calving on the performance, metabolism, and immunity of 28 dairy calves during their first month of life. Blood samples were collected from 28 Holstein cows at calving. Median results for maternal variables including non-esterified fatty acids (NEFA), β-hydroxybutyrate (BHB), glucose, total protein (TP), albumin, triglycerides (TG), total cholesterol (TC), haptoglobin (Hp), body weight (BW), and body condition score (BCS) were determined. These median values served as a basis for categorizing the offspring into two groups based on their dams’ high or low degree of each maternal variable. Additionally, calves were categorized by the season of birth (Spring vs. Winter), with 14 in each. Blood samples were collected from the calves at birth and on days 1, 7, 14, and 28 to assess IgG, biochemical parameters, and haptoglobin concentration. Reactive oxygen species (ROS) production by polymorphonuclear cells stimulated by various agents was also evaluated. Clinical assessments were conducted for diarrhea and bovine respiratory disease frequencies. Despite the overall health of the cows, differences were observed in the calves between maternal groups. Heavier cows with high maternal BCS tended to have larger offspring, while high maternal BCS was associated with increased diarrhea prevalence. Low maternal BCS resulted in a stronger innate immune response, indicated by higher ROS production. Calves from cows experiencing metabolic changes during calving displayed elevated Hp concentrations. Spring-born calves were larger but had lower serum IgG concentration and reduced innate immune response compared to winter-born calves. Additionally, spring-born calves exhibited higher Hp and increased diarrhea prevalence on day 28. These findings underscore the importance of the prenatal period in determining neonatal health and suggest further research to elucidate the long-term clinical implications of maternal effects on offspring health and growth. Investigating offspring constituents later in life can provide insight into the persistence of maternal effects over time.

## Introduction

Studies have shown that events during pregnancy influence fetal development and can affect the newborn throughout its life. This phenomenon is called fetal programming, and changes, as a result of maternal interactions, may reflect alterations in the offspring’s health and productivity (1–3).

In cattle, the classical model of maternal stress during the prepartum period is exposure to environmental heat conditions. Cows that experience heat stress give birth to calves that are 5 kg lighter than those born to dams that did not experience heat stress (4). Dahl et al. (5) demonstrated late gestation heat stress results in calves with lower body weight at birth, shorter stature at weaning, and failure to achieve the same weight or height at 12 months of age compared with calves from dams that were cooled when entered in the dry period. Studies have also shown that maternal heat stress generates changes in the immune system, with lower proliferation and response of immune cells (4, 6–8) and impairment of passive immunity transfer (4, 9). Furthermore, Monteiro et al. (10) demonstrated that the effects of *in-utero* exposure to heat stress are detrimental to milk yield and reproductive performance, these effects extend to at least the first lactation of offspring.

Laporta et al. (11) found that heat stress in late gestation, in addition to affecting the calf of these cows, also affected the granddaughters, having transgenerational effects, and decreasing milk production and survival rates (12). In this way, prenatal conditions have the potential to affect the productivity and health status of replacement heifers, and seasonality can generate differences as a result of different weather conditions, mainly related to the temperature to which the animals are exposed (1).

Despite the large number of publications highlighting the importance of metabolic stress during the transition period for dairy cows, there is limited information on the effect of maternal metabolism on calves’ health. The transition period is characterized by metabolic stress due to excessive lipid mobilization, oxidative stress, and inflammatory dysfunction (13), when the cow needs to adapt to the increase in nutrient requirements associated with fetal growth, colostrogenesis and lactogenesis (14). Ling et al. (15) demonstrated that maternal metabolic stress during the final stage of pregnancy has altered the immune response and metabolic profile of calves in the first month of life, with calves born from cows with high concentrations of NEFA having lower body weight at birth. A pro-inflammatory profile was found in calves exposed to high concentrations of maternal NEFA. In addition, to substantial influence on the dam, maternal stress during late gestation also affects the fetus, and prenatal stress also exerts carryover effects on the offspring in postnatal life (16, 17).

It remains unknown whether other maternal factors during late gestation have similar detrimental effects on neonatal calves’ health, performance, and immunity. This research hypothesized that maternal factors during late gestation influence the health, metabolism, and immunity of calves during the neonatal period. Therefore, the general objective was to evaluate the impact of maternal variables on the calving day on the performance, metabolism, innate immune response, and disease occurrence presented by Holstein’s calves during the neonatal period. The specific aims of this study were to verify diarrhea and Bovine Respiratory Disease prevalence of calves; to check the performance of neonates by measuring thoracic perimeter, withers height, and rump width; to analyze the innate immune response and inflammatory profile in calves by measuring haptoglobin, total protein, the production of neutrophils’ reactive oxygen species challenged by *Staphylococcus aureus*, *Escherichia coli*, and *S. hyicus*. Besides that, all the analytes were compared in two seasons: spring and winter.

## Materials and methods

### Animals and farm conditions

The Animal Research Ethics Committee of the School of Veterinary Medicine and Animal Science of the University of São Paulo approved all procedures involving animals in this study (Protocol Number 6740260218). This research was performed between July and November 2018 in a commercial dairy farm located in Descalvado, São Paulo, Brazil, latitude 21°57′44.9″S and longitude 47°41′44.8″W. Twenty-eight multiparous Holstein dairy cows between 2nd to 5th lactation were evaluated, excluding dams that calved males, stillbirths, twins and neonates with low vitality immediately after birth, screening by <7 Apgar score (18), adapted by Lange et al. (19). The cows that produced a minimum volume of 3 L of colostrum with more than 21% BRIX were included.

The cows were dried off at approximately 220 days of gestation (dried period 73 ± 29.9 days). At dry-off, cows received an intramammary treatment with a long-acting antimicrobial (Cepravin^®^ Dry Cow, MSD Animal Health) to prevent mastitis and an internal teat sealant (Teatseal^®^, Zoetis). To improve protection against neonatal diarrhea, cows were vaccinated with a single dose of an inactivated combination of bovine rotavirus, bovine coronavirus, and *E. coli* F5 (K99). To improve protection against neonatal diarrhea, cows were vaccinated with a single dose of an inactivated combination of Bovine Rotavirus, Bovine Coronavirus, and *E. coli* F5 (K99) (Rotavec^®^, MSD Animal Health), besides one dose of vaccine against mastitis composed by *E. coli* J5 bacterin and toxoid (J-VAC^®^, Boehringer-Ingelheim). Dams also received two doses of commercial vaccines against Bovine Respiratory Disease (BRD) in the dry period (Cattle Master Gold FP5/L5^®^, Zoetis). The dams were transferred to the indoor compost-barn, collective maternity housing with cross-ventilation 30 days before the expected calving date.

Between 60 and 30 days from the expected date of calving, the animals received Diet A and Concentrate A, while between 30 days from the expected date of calving until the real-time of calving the cows received Diet B and the Anionic Concentrate (Supplementary Tables S1, S2). The total mixed ration (TMR) diets were offered twice a day, considering the intake of 13–14 kg of dry matter (DM) per animal. Minerals and vitamins were added in the manufacture of concentrate and mineral salt and water were provided *ad libitum*. The feed corroborated or exceeded nutrients and energy requirements for pre-calving dairy cows (20).

The calf’s management from immediately after birth until 28 days of life was described by the same authors of this present study (21). The dams were milked immediately after calving using a portable milking machine at the maternity pen. The first colostrum feeding was provided using a 3 L bottle offered within an hour after delivery. The mean of colostrum ingested by the calves was 3.71 ± 1.05 L during the first 18 h, with a maximum and minimum volume ingested was 6.5 L and 2.0 L, respectively. The colostrum was screened according to the quality using a digital Brix handheld refractometer (MISCO DD-2 Refractometer, Misco^®^).

To prevent umbilical infection between birth and 10 days of life approximately, the external components of the umbilical stump of the animals were dipped in an antiseptic commercial based on dichlorvos, picric acid, and iodoform (Umbicura^®^, Umbicura, Brazil).

After the birth until the 14th day, the calves were housed in closed-sided individual housing. Water and starter produced on farm *ad libitum* without probiotics were introduced from the 2nd and 3rd day of life, respectively. The average starter intake was 100 g per day per animal during the first 14 days. From the 15th to the 28th day of life, the calves were housed in closed-sided individual housing with a bed of hay (21).

### Sampling procedures

At calving, the body condition score (BCS) of each dam was performed by the same evaluator, using the BCS composed of a scale from 1 to 5 with intervals of 0.25 (22), and the weighing was carried out through measurement of the thoracic perimeter, with weighing tape for bovine. After milking, within a maximum of 1 h after calving, blood samples were collected via coccygeal venipuncture by using two plain vacuum tubes with fluoride sodium and without anticoagulant to obtain aliquots of plasma and serum, respectively.

Calves were blood sampled via jugular venipuncture during the first month of life, being immediately after birth (first day of life—D1) and D2 (1.89 ± 0.74 day), D7 (6.11 ± 1.37 day), D14 (13 ± 1.47 day) and D28 (28.11 ± 1.29 day) days of life. Sampling time was kept consistent throughout the study, with calves being sampled 2 h before the morning milk feeding. The blood was collected into two plain vacuum tubes containing fluoride sodium and no anticoagulant to obtain plasma and serum, respectively. In the sampling time, calves were given a general physical examination that included: vital signs, hydration status, ocular mucous, capillary refill time, and palpation of lymph nodes (23). Furthermore, fecal and bovine respiratory disease (BRD) scores were assessed following the Calf Heath Scoring Criteria previously published by The University of Wisconsin (Madison, United States) by McGuirk (24). Fecal scores were assigned as 0—normal consistency, 1—pasty, semi-formed; 2—pasty with the largest amount of water; or 3—a liquid with fecal content adhered in the perineum and tail. Calves were assessed as having diarrhea when the scores were 2 or 3. BRD was scored using a combination of the following parameters: rectal temperature, cough, nasal and ocular secretion, and ear position with a score of 0–3 for each based on the severity of each. Calves were assessed as having bronchopneumonia when the total of these scores was ≥4 (25). The umbilical region was evaluated by inspection and palpation to detect inflammation.

Calf weights were estimated with a heart girth tape placed vertically at the point of the elbow. Body measurements including rump width (distance between the points of hook bones) and height at the withers (distance from the base of the front feet to the withers) of the calves were performed during the neonatal period.

### Analytical determinations

The white blood count (WBC) was performed by ADVIA 2120i^®^ hematological analyzer. For samples obtained from dams and calves, the measurements of biochemical biomarkers were performed with specific commercial kits for each analyte performed on an automated biochemical analyzer (Labmax 240, Labtest^®^, Japan). Quantification of total cholesterol (TC—Labtest^®^, 76-2/100, Brazil), triglycerides (TG - Labtest^®^, 87-2/250, Brazil), total protein (TP—Labtest^®^, MG3880, Brazil), and albumin (Labtest^®^, 19-1/250, Brazil) was performed from serum samples, and the quantification of glucose (Labtest^®^, 133-1/500, Brazil), non-esterified fatty acids (NEFA—Randox^®^, FA115, United Kingdom) and β-Hydroxybutyrate (BHB—Randox^®^, RB1007, United Kingdom) were obtained from fluoride plasma samples.

The concentration of haptoglobin (Hp) was determined based on its ability to bind to hemoglobin (Hb), using spectrophotometry, as described by Ramos et al. (26). Serum IgG concentrations were quantified using an in-house sandwich ELISA according to the procedure described by Gomes et al. (27), according to previously published by Reber et al. (28).

### Assessment of reactive oxygen species

At first, 3 mL of blood was added to silicon sterile tubes of 15 mL. Initially, the blood was lysed by adding 6 mL of Tris-chloride ammonia lysis solution, incubated at 37°C for 30 min. Posteriorly, the tubes were centrifuged in 290 × g for 10 min, afterwards, the cells were washed in 5 mL of salt-buffered solution twice at 290 × g for 5 min. After the last washing, the supernatant was discarded and the leukocytes were diluted in 1 mL of RPMI-1640 cell culture medium without red phenol (Cat No.: 7509, Sigma-Aldrich, St Louis, MO) supplemented with 10% of fetal serum inactivated by heat and 2 mM L-glutamine (Cat No.:21051-024, Gibco^®^, Brazil).

The concentration and viability of leukocytes was determined by Trypan Blue exclusion. 50 μL of cell suspension was diluted in 450 μL of Trypan Blue 0.04%. From this mixture, 10 μL was pipetted in the Newbauer chamber, counting the number of living and dead cells in a quadrant of the chamber specific for counting leucocytes. Finally, the cell suspension was adjusted to 3 × 10^6^ cells/mL.

The leukocyte suspension (100 μL) was plated in flat bottom cell cultivation plates, being afterward diluted in 100 μL of supplemented cell cultivation medium (non-stimulated cells) or a cell cultivation medium containing specific treatment (stimulated cells). The cells were stimulated with inactivated antigens like *Staphylococcus aureus* (pure); *Escherichia coli* (pure); *S. hyicus* (pure) and Phorbol Myristate Acetate at 10-6 M (PMA—P8139, Merck Millipore, Darmstadt, Germany, Sigma). The bacteria dilutions used were determined in previous studies (30) in which the maximum production of reactive oxygen species (ROS) production after the incubation of leukocytes with the stimulus. Finally, 10 μL of subtract DHR-123 (dihydrorhodamine 123) with a final concentration of 10 μM was added in each well, except for the negative control containing only the medium (medium background).

The plates were incubated for 2 h in a CO_2_ incubator at 37°C. The fluorescent reading was performed in Fluoroskan Ascent FL (Thermo Fisher Scientific, Massachusetts, United States), excitation 485 nm and emission 538 nm. The fluorescence was obtained with the average rate of fluorescence unit (AFU). In each test, the PMA was used as a maximum positive control and the RPMI-1640 medium was used as a negative control. The ROS production in the concentration of each stimulus was presented as a response ratio (RR), calculated as follows: AFU value of stimulated cells/ AFU value of unstimulated cells.

### Maternal factors and groups classification

The maternal analytes BCS, NEFA, BHB, glucose, total protein, albumin, triglycerides, cholesterol, Hp, and body weight (BW) were considered as quantitative independent variables. The experimental groups were established according to the method used by Ling et al. (15), so it was calculated the medians for each maternal metabolic biomarker to set offspring’s data in low and high groups. In cases where the maternal result was equal to the median, the offspring was allocated to the low group (Table 1).

**Table 1:** Median (cut-off values) of maternal biomarkers used to set the offspring in experimental groups.

Maternal factors	Median	Groups
NEFA	0.53 mmol/L	High *n* = 14
		Low *n* = 14
BHB	0.42 mmol/L	High *n* = 13
		Low *n* = 15
Glucose	89.5 mg/dL	High *n* = 14
		Low *n* = 14
TP	6.2 g/dL	High *n* = 14
		Low *n* = 14
Albumin	2.75 g/dL	High *n* = 14
		Low *n* = 14
TG	30.70 mg/dL	High *n* = 14
		Low *n* = 14
TC	68.5 mg/dL	High *n* = 13
		Low *n* = 15
Hp	23.11 mg/dL	High *n* = 13
		Low *n* = 15
BW	758 kg	High *n* = 14
		Low *n* = 14
BCS	3.0	High *n* = 15
		Low *n* = 13

NEFA, non-esterified fatty acids; BHB, β-hydroxybutyrate; TP, total protein; TG, triglycerides; TC, total cholesterol; Hp, haptoglobin; BW, body weight; BCS, body condition score.

In addition, the calves were also distributed by qualitative variables: the season of the birth, considering Winter (14 calves born in July and August) and Spring (14 calves born in September and October). During the experiment, weather data (minimum, maximum, and mean temperatures) was collected from the AccuWeather database for the region of Descalvado, São Paulo state, Brazil. During July and August, the average maximum temperature was 28.2°C; the average minimum temperature was 9.2°C and the average thermal amplitude was 21.2°C; while during September and October the average maximum temperature, average minimum temperature, and average thermal amplitude were 29.3°C; 15.9°C and 13.4°C, respectively.

### Statistical analysis

The statistical analysis was performed using the SAS (SAS System for Windows, Institute Inc., Cary, NC, United States, version 9.4). Non-parametric quantitative variables were submitted to inverse, log, or square root transformation. Within each maternal group, quantitative variables (BCS, NEFA, BHB, glucose, TP, albumin, TG, TC, Hp, and BW) were compared to determine maternal profiles (low and high). A t-test compared adult cow data between groups. To evaluate the effects of maternal factor (independent variables), time, and group × time interaction, the mixed linear model was used for all data, including the RR of ROS production by PMN cells. Data distribution was checked using the Kolmogorov–Smirnov test with a level significance of 5%. The group and time factors were inserted into the linear model as fixed effects, and the subject (animal) factor as a random effect. The autoregressive (AR), symmetric component (SC), and unstructured (UN) covariance structures were tested based on the Akaike Information Criteria, and therefore the matrices with the lowest values were chosen. The EMEANS command with the Bonferroni test allowed comparisons of the main effects to be obtained. The ordinal qualitative clinical parameters were transformed into qualitative response variables (yes and no), with results expressed in positive and negative frequencies, the differences between groups being determined by the chi-square test. Statistical differences were significant at *p* ≤ 0.05.

## Results

**Figure 1:**
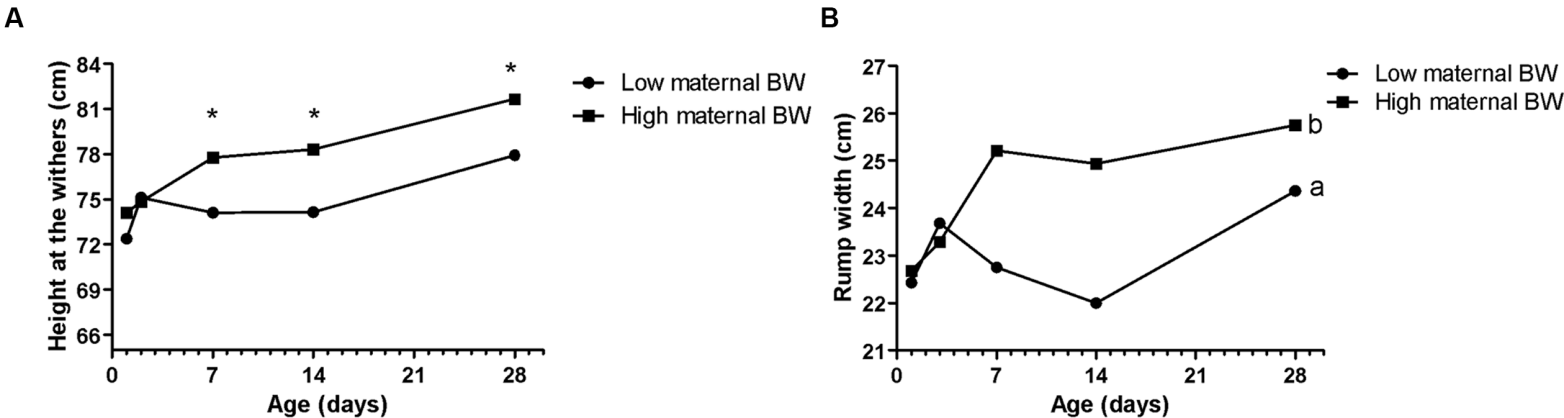
Effects of maternal body weight (BW) on Holstein calves’ parameters – the height of the withers **(A)**, rump width **(B)** during the neonatal period according to maternal groups (low or high - median 758 kg). a, b different letters group effect, *p* < 0.05. *Interaction group × time, *p* < 0.05.

**Figure 2:**
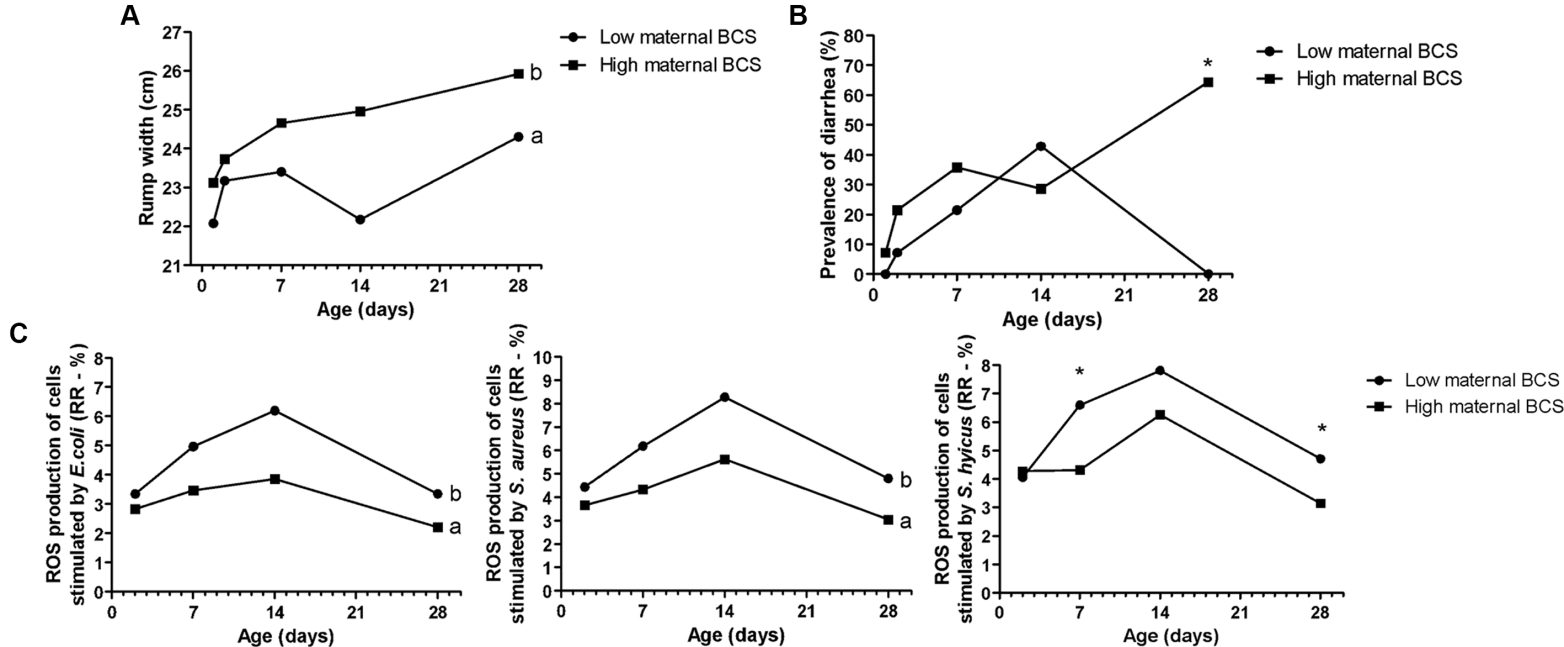
Effects of maternal body condition score (BCS) on Holstein calves’ values—rump width **(A)**, prevalence of diarrhea **(B)**, response ratio (RR) of reactive oxygen species (ROS) production stimulated by *E. coli*, *S. aureus*, and *S. hyicus* **(C)** during the neonatal period according to maternal groups (low or high—median 3.0). ^a,b^Different letters group effect, *p* < 0.05. ^*^Interaction group × time, *p* < 0.05.

**Figure 3:**
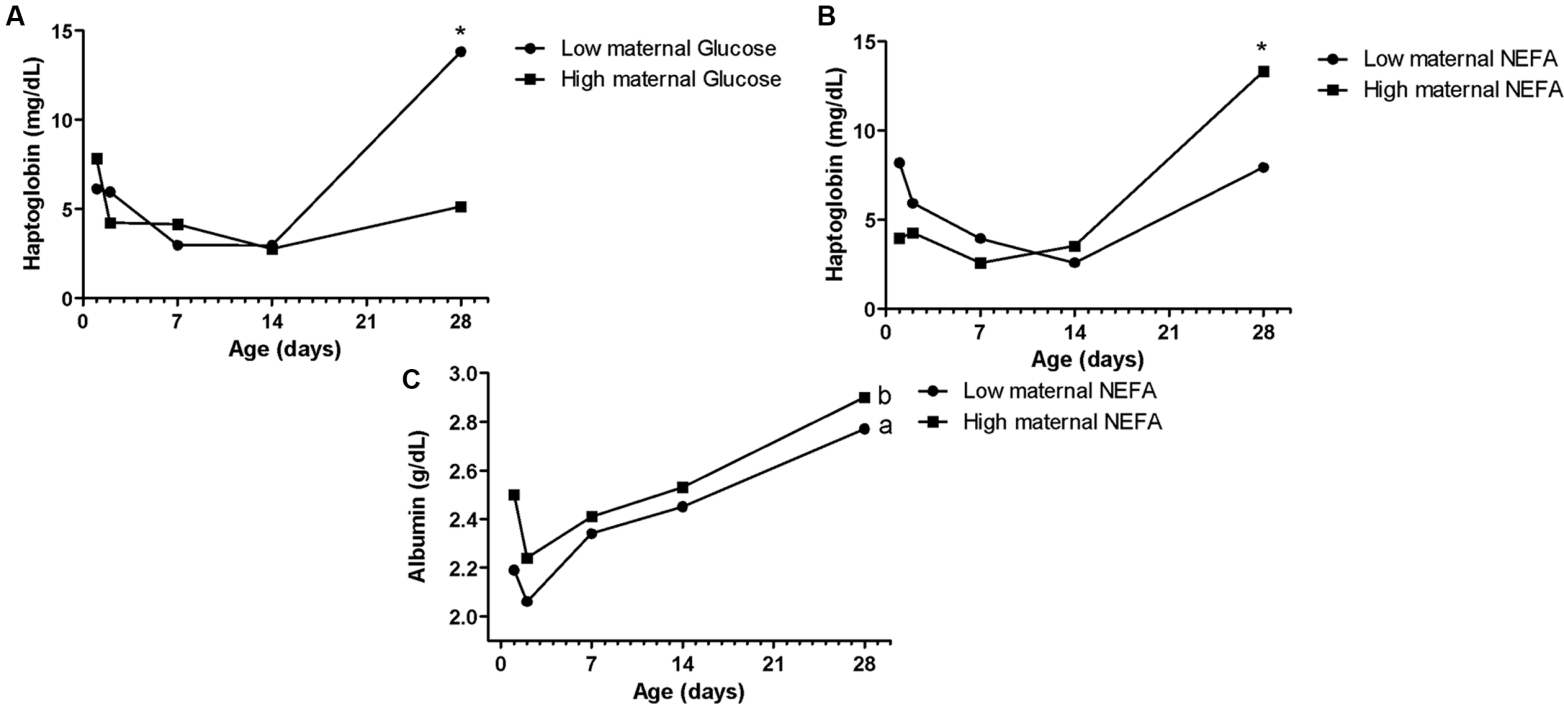
Effects of maternal blood glucose **(A)**, and NEFA **(B,C)** on Holstein calves’ haptoglobin and albumin concentrations during the neonatal period according to maternal groups (low or high—median 89.5 mg/dL for glucose, 0.53 mmol/L for NEFA). ^a,b^Different letters group effect, *p* < 0.05. ^*^Interaction group × time, *p* < 0.05.

**Figure 4:**
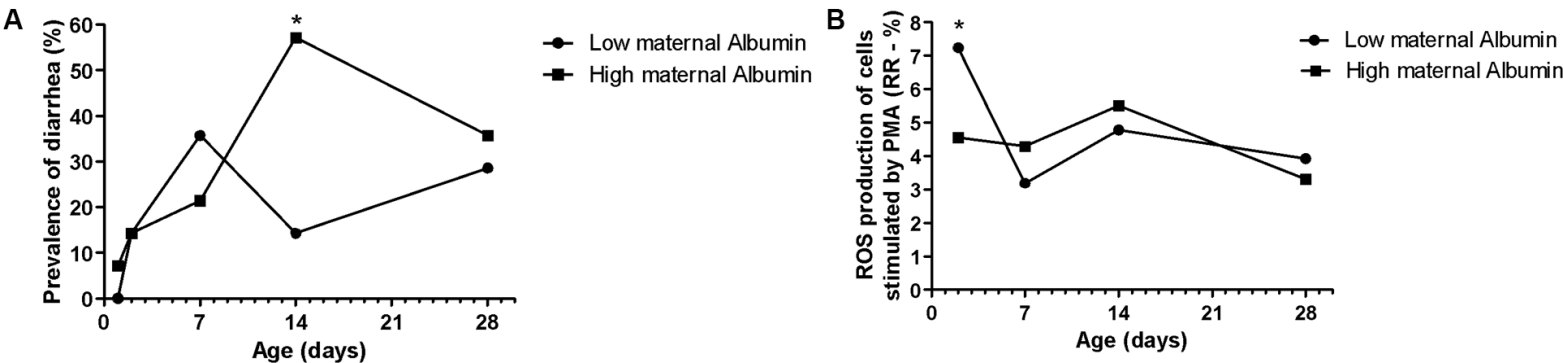
Effects of maternal albumin on Holstein calves’ values—prevalence of diarrhea **(A)**, and response ratio (RR) of reactive oxygen species (ROS) production of cells stimulated by PMA **(B)** during the neonatal period according to maternal groups (low or high—median 6.2 g/dL). ^*^Interaction group × time, *p* < 0.05.

**Figure 5:**
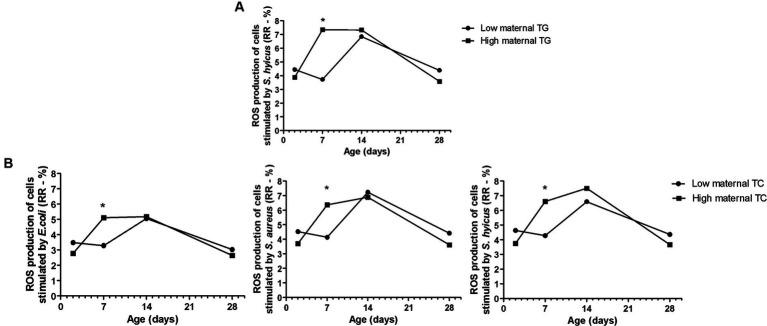
Effects of maternal Triglycerides (TG - **A**) and Total Cholesterol (TC - **B**) on Holstein calves’ parameters (response ratio of ROS production of cells stimulated by *E. coli, S. aureus, S. hyicus*) during the neonatal period according to maternal groups (low or high - median 2.75mg/dL for TAG, and 30.70mg/dL for cholesterol). *Interaction group × time, *p* < 0.05.

**Figure 6:**
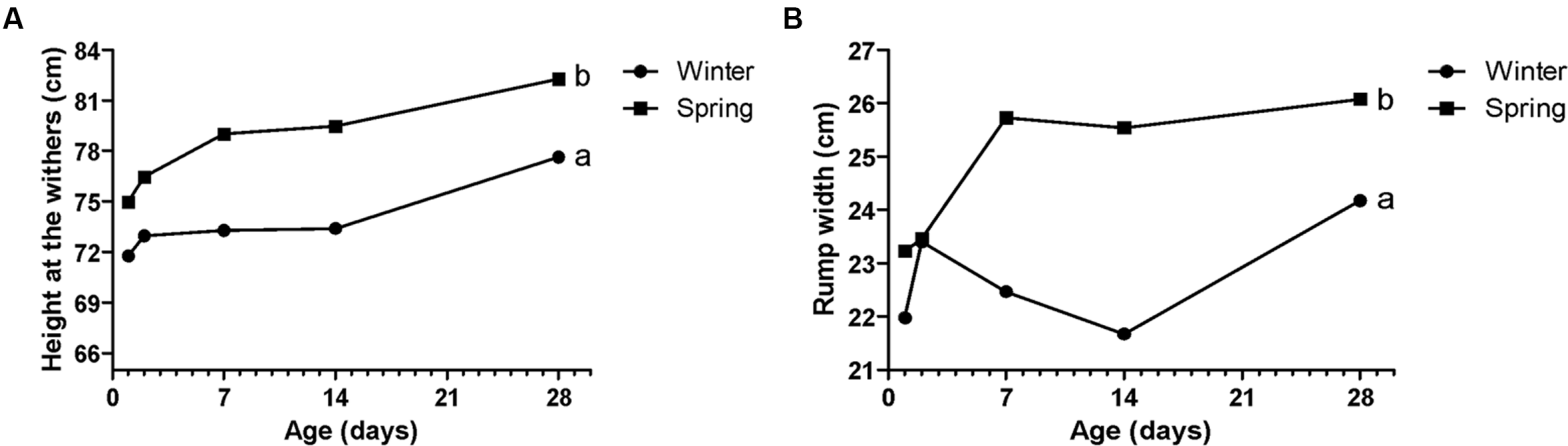
Performance parameters - rump width **(A)** and height of the withers **(B)** of Holstein calves during the neonatal period divided by season (winter x spring). a, b different letters group effect, *p* < 0.05.

**Figure 7:**
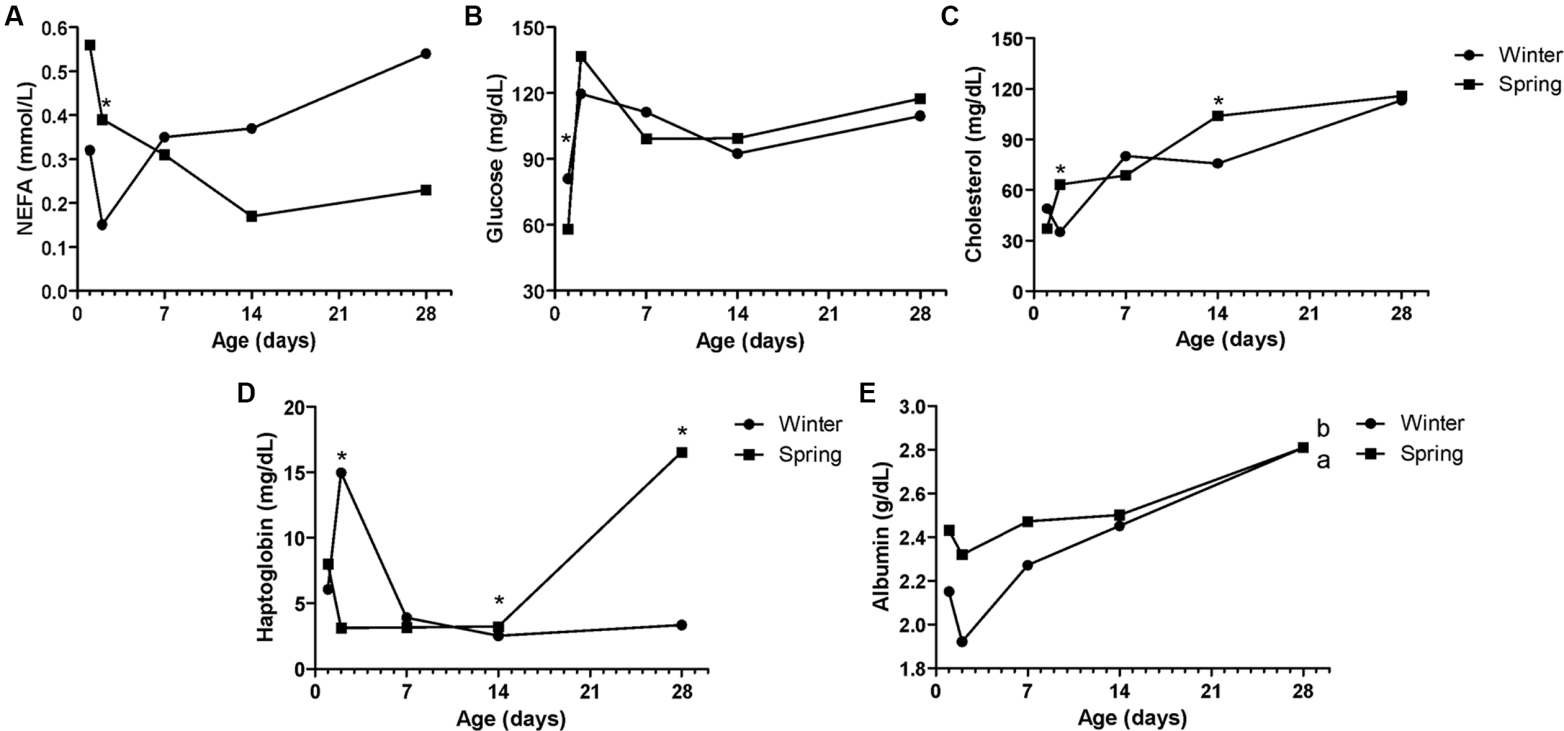
Metabolic constituents—NEFA **(A)**, glucose **(B)**, total cholesterol (TC—**C**), haptoglobin **(D)**, and albumin **(E)** of Holstein calves during the neonatal period divided by season (winter × spring). ^a,b^Different letters group effect, *p* < 0.05. ^*^Interaction group × time, *p* < 0.05.

**Figure 8:**
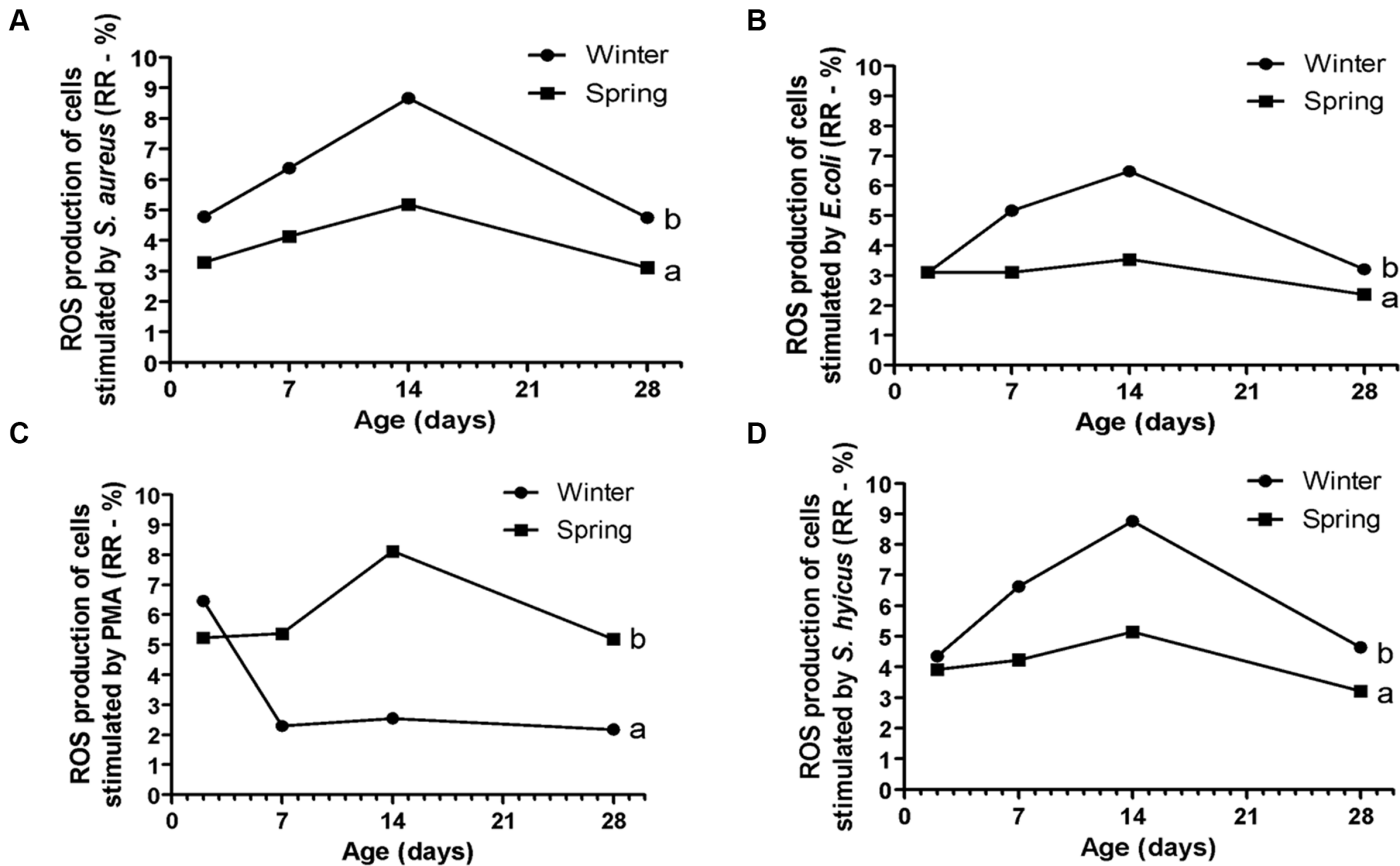
Response ratio (RR) of reactive oxygen species (ROS) of cells stimulated by PMA **(A)**, *E. coli* **(B)**, *S. aureus* **(C)**, and *S. hyicus* **(D)** of Holstein calves during the neonatal period divided by season (winter × spring). ^a,b^Different letters group effect, *p* < 0.05.

**Figure 9:**
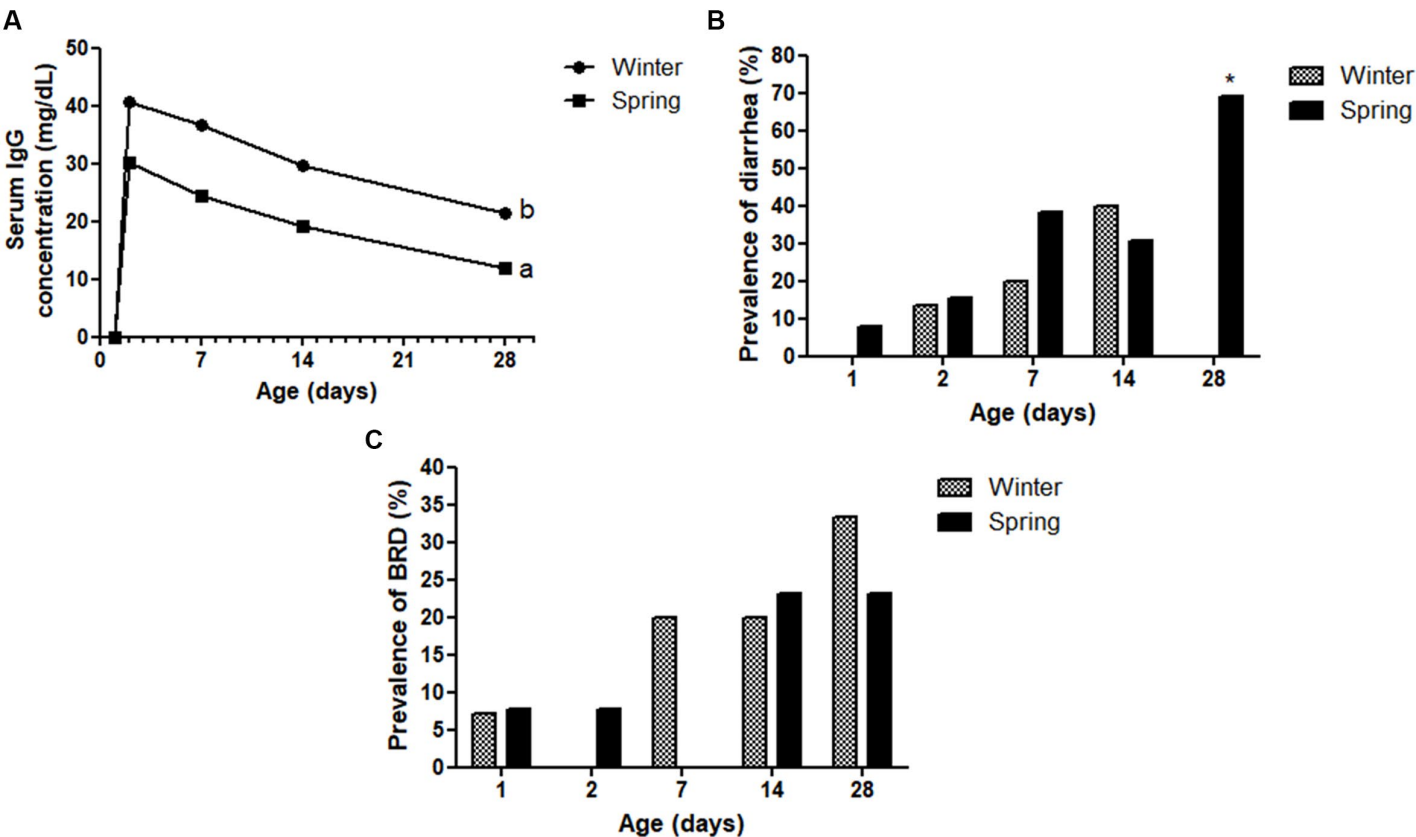
Serum IgG concentration **(A)**, diarrhea **(B)**, and bovine respiratory disease (BRD—**C**) prevalence (%) of Holstein calves during the neonatal period divided by season (winter × spring). ^a,b^Different letters group effect, *p* < 0.05. ^*^Interaction group × time, *p* < 0.05.

The analysis of the effects of maternal constituents, time, maternal group (low and high), and group × time interaction are presented in the Supplementary material, with the *p*-values obtained in each variable (Supplementary Tables S3–S9). Results with statistical differences were presented in Figures 1–5 and Table 2, which show the maternal group and the affected calves’ results. Figures 6–9 and Table 3 exhibited the influence of seasonality on offspring variables. The variation of values of the newborn calves through the time points (D1, D2, D14, and D28) was not discussed, because it was previously published by the same authors (21), except for RR of ROS production. Maternal BHB, TP, and Hp had no group effect and no interaction group × time.

**Table 2:** Metabolic parameters of the Holstein calves during the neonatal period according to different maternal groups (low and high).

Calf parameter	Maternal group	D1	D2	D7	D14	D28	Group	Time	Interaction G × T
GLU (mg/dL)	High BCS	61.77 ± 29.48	145.60 ± 29.80^a^	99.92 ± 15.39	97.08 ± 8.02	113.31 ± 12.53	0.8672	<0.0001	0.0339
	Low BCS	77.57 ± 24.82	113.50 ± 19.35^b^	110.73 ± 20.17	94.60 ± 23.86	113.27 ± 14.49			
BHB (mmol/L)	High TG	0.01 ± 0.01^A^	0.02 ± 0.03^A^	0.04 ± 0.05^A^	0.04 ± 0.04^A^	0.03 ± 0.03^A^	0.0240	0.1883	0.1365
	Low TG	0.07 ± 0.10^B^	0.04 ± 0.04^B^	0.08 ± 0.14^B^	0.01 ± 0.01^B^	0.06 ± 0.04^B^			
NEFA (mmol/L)	High Alb	0.55 ± 0.36^A^	0.27 ± 0.20^A^	0.29 ± 0.14^A^	0.39 ± 0.64^A^	0.60 ± 0.94^A^	0.0258	0.5132	0.2228
	Low Alb	0.32 ± 0.26^B^	0.26 ± 0.25^B^	0.37 ± 0.38^B^	0.17 ± 0.04^B^	0.19 ± 0.07^B^			
	High TP	0.56 ± 0.38^A^	0.33 ± 0.28^A^	0.41 ± 0.38^A^	0.22 ± 0.09^A^	0.59 ± 0.95^A^	0.0200	0.5175	0.2730
	Low TP	0.31 ± 0.22^B^	0.19 ± 0.12^B^	0.25 ± 0.11^B^	0.33 ± 0.65^B^	0.20 ± 0.06^B^			
	High TC	0.53 ± 0.36^A^	0.26 ± 0.19^A^	0.38 ± 0.38^A^	0.36 ± 0.62^A^	0.56 ± 0.92^A^	0.0175	0.5764	0.6229
	Low TC	0.32 ± 0.25^B^	0.27 ± 0.26^B^	0.27 ± 0.10^B^	0.18 ± 0.03^B^	0.21 ± 0.06^B^			
	High TG	0.50 ± 0.37^A^	0.23 ± 0.13^A^	0.37 ± 0.40^A^	0.38 ± 0.64^A^	0.59 ± 0.95^A^	0.0401	0.5110	0.3331
	Low TG	0.37 ± 0.29^B^	0.30 ± 0.29^B^	0.29 ± 0.10^B^	0.17 ± 0.05^B^	0.19 ± 0.06^B^			
TC (mg/dL)	High NEFA	54.56 ± 41.75	44.43 ± 22.93	60.74 ± 20.72^b^	104.66 ± 32.11^a^	110.28 ± 29.08	0.7710	<0.0001	0.0120
	Low NEFA	39.20 ± 16.37	49.74 ± 28.44	80.38 ± 20.21^a^	82.61 ± 21.97^b^	115.85 ± 27.99			
	High GLU	49.74 ± 34.93	45.32 ± 25.14	79.79 ± 22.33	78.76 ± 22.59^b^	120.82 ± 32.16	0.7147	<0.0001	0.0451
	Low GLU	37.44 ± 11.5	51.12 ± 28.76	69.74 ± 21.08	99.06 ± 27.23^a^	107.69 ± 22.06			
	High BW	43.92 ± 33.97	63.19 ± 30.32^a^	67.66 ± 24.01	97.40 ± 28.93	118.04 ± 22.24	0.1016	<0.0001	0.0121
	Low BW	43.26 ± 16.71	33.25 ± 8.70^b^	81.86 ± 17.68	80.42 ± 21.96	110.48 ± 32.99			

NEFA, non-esterified fatty acids; BHB, β-hydroxybutyrate; TP, total protein; TG, triglycerides; TC, total cholesterol; Hp, haptoglobin; GLU, glucose; BW, body weight.

**Table 3:** Mean ± standard deviation of the maternal variables categorized by the season at calving time.

Maternal variables	Winter	Spring
NEFA (mmol/L)	0.47 ± 0.22	0.69 ± 0.27^*^
BHB (mmol/L)	0.33 ± 0.13	0.42 ± 0.19
Glucose (mg/dL)	107.00 ± 28.41	82.69 ± 22.26^*^
TP (μmol/L)	6.16 ± 0.69	6.04 ± 1.76
Albumin (g/dL)	2.71 ± 0.20	2.56 ± 0.42
TG (g/dL)	33.37 ± 14.43	31.79 ± 20.22
TC (mg/dL)	72.43 ± 16.65	69.82 ± 21.30
Hp (mg/dL)	23.55 ± 7.74	25.32 ± 11.75
BW (kg)	693.13 ± 86.70	796.85 ± 52.33^**^
BCS (1–5)	2.85 ± 0.43	3.42 ± 0.26^**^

^*^p < 0.05 and ^**^p < 0.01.NEFA, non-esterified fatty acids; BHB, β-hydroxybutyrate; TP, total protein; TG, triglycerides; TC, total cholesterol; Hp, haptoglobin; BW, body weight; BCS, body condition score.

Maternal body weight (BW) affected calf performance, with calves born from heavier cows having a greater height at the withers (Figure 1A), with interaction group × time on D7, D14, and D28. While for rump width, there was only a group effect, in which high cow’s BW resulted in larger calves from D7 to D28 (Figure 1B). Similarly, the high maternal BCS resulted in higher rump width of calves during the neonatal period (Figure 2A). This high factor affected the prevalence of diarrhea in the calves, which was higher in D28 (Figure 2B). The low BCS of cows influenced the higher RR of ROS production of PMN cells stimulated by all the antigens (Figure 2C). Values of BCS above 3 of cows and heavier dams also influenced the higher calf’s glucose concentration and TC, respectively, at D2 (Table 2).

The maternal low glucose (Figure 3A) and high NEFA resulted in high Hp concentration (Figure 3B) in their offspring on D28, as well as high NEFA influenced high albumin during all the sample times (Figure 3C). The albumin maternal factor affected the prevalence of diarrhea (Figure 4A) and RR of ROS production of PMN stimulated by PMA (Figure 4B). The high albumin of cows resulted in a high prevalence of diarrhea on D14. However, the low albumin group affected higher RR of ROS production (challenged by PMA) on D2. The high maternal glucose resulted in lower values of the calf’s TC on D14 (Table 2). The high NEFA maternal group affected lower values of calf’s TC on D7 and higher values on D14 (Table 2).

The high maternal TG and TC influenced the higher RR of ROS production of PMN challenged by *E. coli*, *S. aureus*, and *S. hyicus* in calves, particularly on D2 (Figure 5). The biochemical maternal factors such as TG, TC, TP, and Albumin resulted in a group effect only for the NEFA concentration of the offspring, which the high group influenced in higher values (Table 2). The same happened for the TG maternal group and BHB calf’s concentration.

Spring calves had a higher weight of the wither and rump width (Figure 6). Seasonality also influenced NEFA; glucose; TC; Hp; albumin (Figure 7); RR of ROS production by PMN cells (Figure 8); IgG; and prevalence of diarrhea of calves (Figure 9B). Initially (D2) winter calves had higher Hp concentration, and on D14 and D28 the values were higher in spring calves. The winter calves had greater IgG concentration (Figure 9A), higher RR of ROS production stimulated by *S. aureus*, *S. hyicus*, and *E. coli*, and a higher prevalence of diarrhea on D14. The season affected the frequency of diarrhea on D28, in which spring calves showed more prevalence than winter. Regarding the ROS production by PMN cells, the behavior after stimulation by *E. coli*, *S. aureus*, and *S. hyicus* were very similar for winter and spring calves, throughout the sample times. A peak was observed on D14 for all bacteria. Although without a seasonal effect, the frequency of BRD is shown in Figure 9C, in which a higher prevalence in winter on D28 can be observed.

The comparison of maternal factors between the seasons showed differences in NEFA, glucose, BW, and BCS. Cows during the spring had higher NEFA, BW, and BCS, however, they had lower glucose (Table 3).

## Discussion

### Maternal effects

The effect of maternal factors on the values of Holstein newborn calves was evaluated, in addition to the seasonal effect. Thus, two groups were established according to the median of maternal values for each factor, as Ling et al. (15) did in their research. Although high or low groups were determined, it should be stated that the cows in this study were clinically healthy, even though median values may have been above reference values, such as NEFA >0.4 mmol/L (29).

Metabolic and nutritional factors can influence maternal-fetal nutrition, thereby impacting fetal growth and development. Low birth weight may indicate compromised fetal nutrition. Additionally, this compromise may lead to greater susceptibility to diseases, higher mortality, and poorer productivity throughout the lives of these animals (2).

Calves born from dams with higher BW and BCS exhibited better performance in terms of wither height and rump width after the first week of life. Cows with higher BW and BCS were better equipped to meet the demands of the fetus, resulting in improved development in their offspring compared to calves from cows with a BCS <3.0. It is crucial to emphasize that the designation “high” for cows does not imply that they were overweight; rather, it signifies that they had an appropriate BCS. Considering that BCS serves as an indicator of energetic metabolism, the current data aligns with Gao et al. (31), where animals born from cows without energy nutritional restriction exhibited greater body height and body length.

The maternal-fetal environment plays a significant role in influencing the postnatal growth of calves (32). In the present study, the birth weight of calves was not influenced by maternal factors during late gestation. This aligns with findings by Martin et al. (33), who reported that the availability of maternal energy, characterized by adequate or inadequate nutrition in the final gestation period, did not impact calves’ BW (as measured by thoracic perimeter). While birth weight is a valuable indicator, other parameters can provide insights into fetal development and predict future outcomes for offspring (2). Therefore, differences observed in wither height and rump width may reflect events related to fetal programming.

Calves born to cows with high BCS exhibited higher blood glucose on D2, following colostrum ingestion. This can be attributed to variations in insulin sensitivity and the calf’s ability to utilize or uptake glucose, as maternal factors can induce changes in these metabolic pathways in calves (34). Maternal body composition is intricately linked to nutritional management and feed intake, and it holds significance as dams provide nutrition to the fetus from both dietary intake and body reserves (35). It is plausible that cows with higher BCS supplied more energy to their offspring, justifying the observed results.

Another hypothesis relates to the quality of colostrum produced by cows with varying BCS. Previous studies have concluded that maternal nutritional status affects colostrum quality in ruminants (36, 37). Therefore, cows with lower BCS may have produced colostrum with lower energy density, potentially influencing the blood glucose concentration of calves on D2 due to the consumption of their dams’ colostrum.

Moreover, numerous dairy herds have reported a deficiency in colostrum production by cows during the fall and winter seasons (38). This limitation hinders farms from providing sufficient high-quality colostrum to all their calves. Unfortunately, there is a lack of substantial evidence concerning the factors that influence the volume of colostrum produced by dairy cows. In a study conducted by Mann et al. (39), no statistically significant differences in colostrum volume were observed among various dietary planes of energy treatments. However, cows on an energy-controlled diet tended to produce numerically less colostrum than those on a high-energy diet. Taken together, these findings suggest that the nutritional status of the cow before calving can impact colostrum production. The interplay between prepartum diet, colostrum composition and yield, and the health and development of calves warrants further investigation (1).

The changes in biomarker profiles in calves starting after colostrum feeding indicate maternal effects in the colostrum and the calf’s response to those components. Colostrum contains proteins, essential and nonessential amino acids and fatty acids, lactose, vitamins, and minerals, as well as, non-nutrient substances, such as immunoglobulins, and others (40, 41). Therefore, maternal factors may have influenced the composition of colostrum, affecting the different calf profiles found in this study.

Within the metabolic profile, concentrations of glucose, TC, NEFA, and BHB are related to the energy metabolism of ruminants, and changes in their concentrations may indicate different productive levels in these animals (42). TG and TC results reflect the amounts of circulating lipoproteins, composed of lipids absorbed from the diet or synthesized by the body itself (43). The NEFA and BHB concentrations are indicators of lipomobilization, with an increase in their concentrations in states of negative energy balance (3). These analytes were evaluated in experiments with calf diets, indicating their metabolic capacity and reflecting the animal’s response to nutritional differences, which depends on the nutrients absorbed and their utilization (44–46). In this study, variations were found in calves’ NEFA, BHB, and TC concentrations, which were influenced by maternal factors, such as BW, NEFA, glucose, and TG results, showing relationships between cow and calf metabolism. Therefore, these changes in the calf metabolism can then influence their future performance, and modify the nutrients use, and weight gain, however, it is not clear how or, or what will be the real impact of these changes found in calves.

Fat metabolism, through fatty acid oxidation, increases rapidly after birth to meet the energy demand of the newborn calf (47, 48). Calves have fat stores at birth and can mobilize them and provide NEFA for energy supply (49, 50). High NEFA concentration in calves often suggests a lack of energy intake or increased energy demands, which can be a sign of metabolic stress and energy deficiency, leading to poor growth and health outcomes (14). In the present study, NEFA concentration was influenced by maternal protein and lipid profile and may be related to fat stores or lipolysis stimuli. While the BHB concentrations in the first few days after birth were low, it is indicated as a marker of lipomobilization in calves (47, 51). In this research, calves with higher BHB concentrations were born from cows with lower circulating TG, potentially due to the influence of maternal metabolism and lipid reserves.

The relationship between maternal glucose levels and calf NEFA levels highlights the importance of adequate maternal nutrition during late gestation for the metabolic health of both the cow and the calf. Ensuring proper maternal glucose concentration can lead to better colostrum quality and energy transfer to the calf, reducing the need for NEFA mobilization and supporting better health and growth outcomes. Effective dairy management practices focusing on maternal nutrition, colostrum management, and calf health monitoring are essential to optimize these outcomes (52).

Hp is one of several acute-phase proteins produced in response to inflammatory changes associated with infection, making it a potentially useful indicator of calf health (53). In the current study, calves that were born from cows in metabolic stress with high NEFA and low glucose had significantly higher Hp at D28, which suggested a more exacerbated inflammatory condition in these animals at the end of the neonatal period. This finding was compatible with research realized by Ling et al. (15), in which high maternal NEFA concentrations were associated with higher inflammation in calves (expressed by Hp concentrations). The current data and those described by Ling et al. (15) draw attention to the fact that this maternal lipomobilization can also reflect on the inflammatory response of the offspring. Besides that, the highest Hp concentration was shown on D28, when the highest prevalence of diarrhea occurred as well. Thus, the inflammation indicator, in that case, might happen for both reasons.

The offspring of cows with higher BCS had a higher prevalence of diarrhea at the end of the neonatal period (D28). High BCS is related to metabolic stress in cows since cows have more tendency to mobilize body fat, which results in higher NEFA and BHB values (42). The BCS determines the greater or lower predisposition to oxidative status at calving, as the loss of BCS in the prepartum period accentuates this status (54). In this way, the metabolism of these animals was distinct from animals with lower BCS, and this may be related to changes in calves, influencing their susceptibility to diseases, and explaining the higher prevalence of diarrhea and can be related to the inflammatory state in the offspring of cows in metabolic stress.

In the present study, the innate immune response of the offspring was evaluated by ROS production of PMN cells stimulated by common bacteria for dairy herds (27, 55). The lower RR of ROS production of PMN challenged by *E. coli*, *S. aureus*, and *S. hyicus* found related to high maternal BCS was interesting. This fact corroborates with high incidence of diarrhea in the same group, especially on D28, as mentioned before. Therefore, this lower immune response can indicate greater susceptibility to infectious diseases, such as diarrhea found in these calves. The dam’s BCS may have exerted epigenetic effects on the development and immune capacity of the calves, reflecting on their health.

Considering that the cows with high BCS were not obese, and had a desirable BCS since they were healthy, the hypothesis to explain the maternal effect on the immune development of its calf is the metabolic stress related to energetic profile during the transition period (1). Although the maternal NEFA did not affect the immune response in our study, high cow’s NEFA influenced the high Hp concentration in calves, which means that these animals were exposed to proinflammatory factors. This exposition was also associated with greater serum concentrations of reactive oxygen and nitrogen species, which resulted in higher values of oxidant status index in the calves throughout their first month of life (1, 15). However, the mechanism by which prenatal exposure to high maternal NEFA concentrations in late gestation leads to an increased oxidant status is unknown.

According to Ling et al. (15), offspring born from cows that underwent excessive fat mobilization (high NEFA) during prepartum had lower immune responses than calves that had lower concentrations of this constituent. Neonatal exposure to this maternal variable may influence the development of diseases in the first month of life. Gao et al. (31) demonstrated the effects of the maternal diet’s energy density influencing calves’ immune function. Cows fed a diet with low concentrations of energy had granddaughters with lower lymphocyte expression and lower cytokine production response, and this maternal group of cows had greater metabolic stress, with higher NEFA and lower blood glucose concentrations (31).

The calves born to cows with higher TC and TG responded more strongly with ROS production against the bacteria stimulus, especially on D7. It is possible to infer that the greater immune response, shown by elevated RR of ROS during the first 14 days of life, protected the calves from environmental challenges. Nevertheless, on D28 the immune response was not so effective in preventing diseases, such as diarrhea.

Morita et al. (30) compared the immune response of newborn and older healthy calves, and the authors described that *E. coli* generated a higher ROS stimulation index in the youngest calves. They correlated this finding to the fact that the most important disease in these newborn animals was diarrhea, and *E. coli* is a major enteric pathogen, which can promote this clinical alteration in young calves.

Another piece of evidence validating the maternal impact on the innate immunity of newly-born calves was explored by Lopreiato et al. (56). These researchers delved into the immune-metabolic status and growth performance of Simmental calves delivered by cows subjected to pegbovigrastim administration 7 days before calving, which is a long-acting recombinant bovine granulocyte colony-stimulating factor. The findings revealed elevated results of total reactive oxygen metabolites and myeloperoxidase in the treated group. Consequently, they postulated that the activation of the cow’s immune system through pegbovigrastim could have affected the immune competence, and growth performance, as well as the equilibrium between oxidant and antioxidant indices in the neonatal calf.

### Seasonality effects

The seasonality influenced the calf performance, in which spring calves had a higher height at the withers and rump width than those that were born in winter. However, when compared to other studies, cows that had heat stress calved smaller offspring, with lower measures, being that heifers that experienced heat stress *in utero* during late gestation were smaller in weight, stature, and size (57). Also, animals with heat stress had lower weight at birth and in the weaning (4). Despite spring being warmer than winter, calves were born larger in spring, it may be that seasonality did not negatively influence these values, since, despite the temperature difference, spring is not the hottest season of the year in Brazil. Therefore, there may not have been a sufficient negative influence of thermal stress as found in other studies.

The comparison of spring and winter groups allows us to note that cows in spring had more tendency to metabolic stress since they had higher NEFA concentration and lower glucose. These cows had higher BW and BCS, which are risk factors for metabolic stress previously discussed in this current research. Consequently, it is possible that in addition to the seasonal effect, it influenced the metabolic condition of the cows and the results found in the offspring.

Before colostrum intake (D1), the seasonality at calving also affected calf glucose concentration, while animals that were born in the spring presented lower blood glucose than calves born in the winter. Glucose during the fetal period depends almost exclusively on placental supply, and there is a linear relationship between maternal and fetal glucose concentrations (58). Thus, as spring cows had lower glycemia, this may have been reflected in the lower glycemia found in calves on D1.

In addition, another possible explanation for the difference in glucose uptake capacity and insulin sensitivity in calves may have influenced their glycemia. Studies have already reported differences in these analytes in calves due to *in-utero* heat stress at the end of pregnancy. Monteiro et al. (59) showed that the offspring of cows that underwent heat stress at the end of pregnancy had faster glucose clearance and lower insulin clearance in glucose tolerance tests, which may indicate differences in glucose uptake by non-insulin-dependent tissues. Besides that, these calves also had lower BW in the weaning time. Moreover, Tao et al. (60) also demonstrated a different glucose uptake rate in animals born from cows that suffered heat stress, and Guo et al. (61) found differences in insulin secretion, highlighting the epigenetic influence on the metabolism of calves.

This difference in glucose availability and use may also influence other metabolic pathways, such as fat mobilization, and releasing NEFA, and these calves with lower glycemia on D1 had more NEFA on D2. Like it was mentioned before, calves have fat stores at birth and can mobilize them and provide NEFA for energy supply (49, 50), and this may be related to these metabolic changes, which calves with lower glycemia mobilized more lipid reserves. In addition, other authors found differences in the hormone concentration and metabolites in calves that were born from cows that underwent heat stress (60, 61), including lipid metabolism, altering NEFA and BHB concentrations (59) in the same way, alterations in cholesterol values, and also in NEFA related to the season were found in this work.

These spring calves had higher cholesterol concentrations at D2 and D14, which can be related to lipomobilization, but it may also be related to the colostrum ingested, which may have different nutritional concentrations depending on the season. The cholesterol value is an important indicator of the nutritional status of preweaning calves, allowing us to evaluate the contribution of milk to the energy supply of these animals, as demonstrated in studies carried out with calves (62).

The interaction between age and the season occurred for Hp on D2, D14, and D28. Initially, winter calves had higher values than spring ones. This fact can be attributed to the climatic conditions that the animals go through during the winter. Calves that were born during the cold months of the experiment went through mild temperatures, which were below the thermoneutral zone so that the activated thermoregulation mechanisms could be activated, with greater energy expenditure (53, 63), promoting a pro-inflammatory state in calves. However, from D14 to D28, the inflammatory condition was more exacerbated in spring animals. There was a higher incidence of diarrhea on D28 for spring animals, thus, these factors may be related.

Despite the calves being housed in individual suspended cages, another noteworthy consideration is the elevated pluviosity index during spring in Brazil. The combination of increased humidity and high temperatures during this season may contribute to the contamination by pathogenic agents, potentially leading to a higher incidence of diarrhea cases (64). Therefore, the higher prevalence of diarrhea in spring-born calves highlights the need for targeted management strategies to mitigate the effects of environmental stressors and enhance calf health. By optimizing colostrum management, improving the calf environment, monitoring health closely, implementing vaccination and biosecurity measures, and ensuring proper nutrition and hydration, dairy producers can better manage the risks and improve the overall health and productivity of spring-born calves.

The transfer of passive immunity (TPI), measured by IgG concentrations, was lower in spring calves, which probably resulted from a lower IgG absorptive capacity by these calves, a fact that has already been reported in other studies (4, 9). The IgG mass ingested by spring and winter calves did not differ statistically, however, the serum concentration differed among the sample times at the first month of life. This finding can be explained by the better colostrum quality of the cows during winter, maybe because they were in more comfortable weather than in springtime. Although spring calves had lower IgG concentration from D2 to D28, it is clear that they improved their serum concentration from birth to a satisfactory range (excellent TPI ≥25 g/L), which means they did not have a failure of TPI (65). In addition, the decrease of IgG results observed from D2 to D28 for both season groups was expected, as Correa et al. (66) described in their study.

The lower IgG concentration in calves was similarly reported by Seyed Almoosavi et al. (67), Ahmed et al. (68), and Davidson et al. (69) in their studies on the effects of heat stress on offspring. These authors confirmed that heat stress altered the small intestine’s capacity to absorb IgG, leading to increased apoptosis levels and a weakened immune system in calves. Therefore, comparing spring and winter in the current study, it can be inferred that cows experienced heat stress during late gestation, impacting IgG absorption.

The evaluation of innate immunity by RR of ROS production of PMN cells challenged by *E. coli*, *S. aureus*, and *S. hyicus* showed a greater response for winter calves. This fact demonstrated less activity of immune response during springtime. It could happen if the calves did not suffer so many environmental challenges to infectious diseases, or if the immune response was not sufficient to maintain health. Considering the high prevalence of diarrhea during the spring on D28, the second hypothesis can be more acceptable. Besides that, another possibility is that the cows calved in spring endured heat stress. Marrero et al. (7) found lower production of IL-6 by the leukocytes of animals that suffered heat stress in intrauterine life. Tao et al. (4) described lower proliferation of immune cells in animals born to cows with heat stress. Strong et al. (8) also provide immunological evidence of altered innate immunity during the first few weeks after birth in calves due to maternal heat stress during late gestation in the first few weeks after birth. These authors reported differences in innate immunity, interactions with pathogens, pathogen recognition molecules, and cytokines production.

The current study reaffirms previously reported findings in the literature regarding maternal effects on neonatal constituents. However, it has also brought forth additional insights that warrant a more thorough evaluation of the pregnant cow in the prepartum period, along with extended monitoring of their offspring. Consequently, further studies are recommended to delve into the intricate effects of the pregnant cow’s metabolism not only during the neonatal phase of the calf but also throughout the rearing phase and into the productive phase of lactation.

Although a retrospective study was conducted on the effect of maternal lipomobilization up to the first lactation of their offspring, many gaps remain (52). These authors focused on the NEFA concentration in cows 7 to 14 days before calving and categorized the calves into physiological and excessive lipomobilization groups based on NEFA levels ≥0.3 mM. Similar to the present study, they found no difference in the occurrence of diarrhea and BRD in pre-weaned calves. Elevated NEFA concentrations affected the first breeding and the birth weight, however, did not impact other factors associated with calf performance. Consequently, these researchers encouraged further studies with additional indicators of lipomobilization and a balanced sample size of cows with and without excessive lipomobilization.

Based on the presented results, the data suggested that prenatal exposure to various maternal metabolic factors and seasonality (Spring vs. Winter) during late gestation affected certain metabolic constituents and innate immune responses in offspring, potentially influencing disease susceptibility and performance. Heavier cows and high maternal BCS tended to calve bigger animals, as indicated by the higher height at the withers and rump width. However, higher BCS was associated with a higher prevalence of diarrhea on D28. Additionally, a low BCS resulted in a higher ROS production, signifying a more robust innate immune response. Furthermore, calves from cows under metabolic stress, characterized by high NEFA and low glucose, exhibited elevated Hp concentrations. Concerning seasonality, spring-born calves were larger (weight at the withers and rump width), yet they displayed lower IgG concentrations and a reduced innate immune response. Moreover, they exhibited a higher Hp concentration and a greater prevalence of diarrhea on D28. These findings were significant as they underscore the notion that a successful neonatal period commences during gestation. Further research is recommended to elucidate the mechanisms by which metabolic analytes during late gestation influence the immune and metabolic responses of offspring. In addition, exploring the calf’s constituents later in life is suggested, making it possible to know the persistence of the maternal effects over time.

## Data availability statement

The datasets presented in this study can be found in online repositories. The names of the repository/repositories and accession number(s) can be found in the article/Supplementary material.

## Ethics statement

The animal studies were approved by the Animal Research Ethics Committee of the School of Veterinary Medicine and Animal Science of the University of São Paulo (Protocol Number 6740260218). The studies were conducted in accordance with the local legislation and institutional requirements. Written informed consent was obtained from the owners for the participation of their animals in this study.

## Author contributions

FS: Conceptualization, Formal analysis, Investigation, Methodology, Writing – original draft. BS: Writing – review & editing. FM: Writing – review & editing. KS: Data curation, Formal analysis, Methodology, Writing – original draft. EP: Writing – review & editing. VG: Conceptualization, Funding acquisition, Project administration, Resources, Supervision, Validation, Visualization, Writing – original draft, Writing – review & editing.
