# Supplementary material for: Effects of dam metabolic profile and seasonality (Spring vs. Winter) on their offspring’ metabolism, health, and immunity: maternal factors in dairy calves’ analytes
Source: Front Vet Sci. 2024 Jul 11;11:1424960. doi: 10.3389/fvets.2024.1424960 (PMC11285391; doi:10.3389/fvets.2024.1424960)
Supplement: Supplementary file 1 [file Table_1.DOCX]

**SUPPLEMENTAL MATERIAL**

**Table S1.** Diet A provided to dams between 60 and 30 days before calving, and Diet B fed dams between 30 days to calving time during the experiment.

|  | Item | Dry matter (%) |
| --- | --- | --- |
| Diet A | Concentrate A | 5.92 |
|  | Soybean meal | 8.33 |
|  | Wheat straw | 7.80 |
|  | Tifton grass | 13.25 |
|  | Corn silage | 64.70 |
| Diet B | Anionic concentrate | 9.06 |
|  | Wheat straw | 12.41 |
|  | Soybean meal | 19.82 |
|  | Corn silage | 58.71 |
|  | Total | 100 |

**Table S2.** Concentrate A provided to dams between 60 and 30 days before calving (recipe for 2,000kg), and Anionic concentrate was fed to dams between 30 days to calving time during the experiment (recipe for 1,000.8 kg).

|  | Item | Quantities (kg) |
| --- | --- | --- |
| **Concentrate A** | Vitamin E | 2.0 |
|  | Corn grain | 1086.0 |
|  | 570 ca (Nutron) * | 510.0 |
|  | Urea | 200.0 |
|  | OmniGen^®^ (Phibro) | 100.0 |
|  | Yeast | 50.0 |
|  | Magnesium oxide | 25.0 |
|  | Mycofix^®^ (Biomin) | 20.0 |
|  | Sel-plex^®^ (Alltech) | 2.0 |
|  | Difly^®^ (Champion) | 1.5 |
|  | Biotin | 1.0 |
| **Anionic concentrate** | NutriCAB^®^ (Kemin) | 25 |
|  | Colina | 15 |
|  | Vitamin E | 1 |
|  | Salus NA ** | 440 |
|  | Corn grain | 425 |
|  | Vitamin AD3 | 0.25 |
|  | OmniGen® (Phibro) | 40 |
|  | Yeast | 25 |
|  | Urea | 15 |
|  | Mycofix^®^ (Biomin) | 7 |
|  | Nexulin^®^ (Pancosma) | 4 |
|  | Biotin | 1 |
|  | Sel-plex^®^ (Alltech) | 1 |
|  | Difly^®^ (Champion) | 1 |
|  | Probios^®^ (Ourofino Saúde Animal) | 0.5 |

***570 ca (Nutron®):** Guarantee levels of the vitamin and minerals: Calcium (min) 180 g/kg; Calcium (max.): 200 g/kg; Phosphor (min.): 20 g/kg; Vitamin A (min.): 450000 UI/kg; Vitamin D(min.): 300000 UI/kg; Vitamin E (min.): 7000 UI/kg; Magnesium (min.): 145 g/kg; Sulfur (min.): 45 g/kg; Manganese (min.): 3000 mg/kg; Zinc (min.): 3000 mg/kg; Cobalt(min.): 63 mg/kg; Copper (min.): 800 mg/kg; Iodine (min.): 35 mg/kg; Selenium (min.): 22 mg/kg; Fluorine (max): 200 mg/kg; Chrome (min.): 60 mg/kg; Sodic monensin (min.): 1500 mg/kg.

**** Salus NA**: Guarantee levels of the vitamin and minerals: non-protein nitrogen (max): 280 g/kg; Calcium (min) 100 g/kg; Calcium (max.): 150 g/kg; Phosphor (min.): 4500 mg/kg; Magnesium (min.): 60 g/kg; Sodium (min): 20 g/kg; Sulfur (min.): 50 g/kg; Zinc (min.): 1350 mg/kg; Cobalt(min.): 25 mg/kg; Copper (min.): 315 mg/kg; Iodine (min.): 35 mg/kg; Chrome (min.): 25 mg/kg; Selenium (min.): 15 mg/kg; Manganese (min.): 1260 mg/kg; Vitamin A (min.): 225000 UI/kg; Vitamin D(min.): 70000 UI/kg; Vitamin E (min.): 3600 UI/kg; Sodic monensin (min.): 600 mg/kg.

**Table S3 -** Relationship between the high and low maternal groups and calf performance of Holstein calves during the neonatal period (result expressed by *P*-value from the mixed linear model).

| Maternal groups | Body weight | | | Height at the withers | | | Rump width | | |
| --- | --- | --- | --- | --- | --- | --- | --- | --- | --- |
|  | Group | Time | Group x time | Group | Time | Group x time | Group | Time | Group x time |
| NEFA | 0.9060 | <0.001 | 0.7520 | 0.4420 | <0.001 | 0.856 | 0.1280 | 0.005 | 0.4130 |
| BHB | 0.9494 | <0.0001 | 0.4485 | 0.0629 | <0.0001 | 0.3097 | 0.0984 | <0.0001 | 0.3708 |
| Glucose | 0.5954 | <0.0001 | **0.0244** | 0.3602 | <0.0001 | 0.8258 | 0.9009 | <0.0001 | 0.4129 |
| TP | 0.7405 | <0.0001 | 0.8543 | 0.8401 | <0.0001 | 0.8405 | 0.9574 | <0.0001 | 0.4471 |
| Albumin | 0.4128 | <0.0001 | 0.4104 | 0.9260 | <0.0001 | 0.2176 | 0.3419 | <0.0001 | 0.1514 |
| TG | 0.8565 | <0.0001 | 0.3558 | 0.7833 | <0.0001 | **0.0509** | 0.2938 | <0.0001 | 0.8308 |
| TC | 0.2791 | <0.0001 | 0.3027 | 0.2762 | <0.0001 | 0.1475 | 0.0805 | <0.0001 | 0.1103 |
| Hp | 0.4563 | <0.0001 | 0.8866 | 0.4975 | <0.0001 | 0.5241 | 0.4932 | <0.0001 | 0.9518 |
| BW | 0.5545 | <0.0001 | 0.3259 | 0.0687 | <0.0001 | **0.0018** | **0.0178** | <0.0001 | **0.0016** |
| BCS | 0.5884 | <0.0001 | 0.5884 | 0.0585 | <0.0001 | 0.0805 | **0.0179** | 0.0071 | 0.1899 |
| Season | 0.5054 | <0.0001 | 0.2256 | **0.0005** | <0.0001 | **0.0494** | **<0.0001** | <0.0001 | **0.0002** |

The mixed linear model was used to evaluate the main effects of group (low and high), time, and group-time interaction.

P<0.05 was adopted for statistical differences.

Abbreviations: NEFA: Non-esterified fatty acids; BHB: β-hydroxybutyrate; TP: total protein; TG: triglycerides; TC: total cholesterol; Hp: haptoglobin; BW: body weight; BCS: body condition score.

**Table S4.** Relationship between the high and low maternal cut-off values for different biomarkers and the energetic metabolism of Holstein calves during the neonatal period (result expressed by *P*-value from the mixed linear model).

| Maternal groups | Calf biochemical parameters | | | | | | | | | | | | | | |
| --- | --- | --- | --- | --- | --- | --- | --- | --- | --- | --- | --- | --- | --- | --- | --- |
|  | NEFA | | | BHB | | | Glucose | | | Triglycerides | | | Cholesterol | | |
|  | G | T | G x T | G | T | G x T | G | T | G x T | G | T | G x T | G | T | G x T |
| NEFA | 0.3580 | **0.010** | 0.4390 | 0.1630 | 0.0690 | 0.4280 | 0.4050 | **<0.0001** | 0.4390 | 0.5500 | **<0.001** | 0.8820 | 0.7710 | **<0.0001** | **0.0120** |
| BHB | 0.6651 | 0.5212 | 0.4138 | 0.4819 | 0.1888 | 0.2158 | 0.8341 | **<0.0001** | 0.0564 | 0.1852 | 0.1053 | 0.7231 | 0.5705 | **<0.0001** | 0.1097 |
| Glucose | 0.9887 | 0.4983 | 0.4321 | 0.3348 | 0.2038 | 0.6610 | 0.6482 | **<0.0001** | 0.5802 | 0.5720 | 0.0906 | 0.1194 | 0.7147 | **<0.0001** | **0.0451** |
| TP | **0.0200** | 0.5175 | 0.2730 | 0.5738 | 0.2097 | 0.8761 | 0.4438 | **<0.0001** | 0.7766 | 0.7258 | 0.0918 | 0.1415 | 0.7957 | **<0.0001** | 0.2933 |
| Albumin | **0.0258** | 0.5132 | 0.2228 | 0.7721 | 0.1789 | 0.0554 | 0.3711 | **<0.0001** | 0.2385 | 0.8399 | 0.0992 | 0.3768 | 0.2365 | **<0.0001** | 0.1083 |
| TG | **0.0401** | 0.5110 | 0.3331 | **0.0240** | 0.1883 | 0.1365 | 0.7238 | **<0.0001** | 0.1884 | 0.7316 | 0.0963 | 0.2648 | 0.9414 | **<0.0001** | 0.1709 |
| TC | **0.0175** | 0.5764 | 0.6229 | 0.4362 | 0.1927 | 0.0789 | 0.4565 | **<0.0001** | 0.2493 | 0.7033 | 0.1077 | 0.8206 | 0.2227 | **<0.0001** | 0.7485 |
| Hp | 0.1774 | 0.4270 | 0.1682 | 0.5419 | 0.2184 | 0.8272 | 0.6592 | **<0.0001** | 0.4364 | 0.7472 | 0.0891 | 0.3558 | 0.4341 | **<0.0001** | 0.4995 |
| BW | 0.8899 | 0.4961 | 0.5679 | 0.5367 | 0.1896 | 0.1781 | 0.1467 | **<0.0001** | 0.0101 | 0.1021 | 0.1020 | 0.5227 | 0.1016 | **<0.0001** | **0.0121** |
| BCS | 0.2219 | 0.9022 | 0.6881 | 0.5882 | 0.6458 | 0.9021 | 0.8672 | **<0.0001** | **0.0339** | 0.8047 | 0.3652 | 0.6322 | 0.4882 | **<0.0001** | 0.6088 |
| Season | 0.4920 | 0.8896 | **0.0528** | 0.6718 | 0.1652 | 0.4186 | 0.7176 | **<0.0001** | **0.0109** | 0.1120 | **0.0490** | 0.0233 | 0.1573 | **<0.0001** | **0.0008** |

The mixed linear model was used to evaluate the main effects of group (low and high), time, and group-time interaction. G = group; T = time; G x T = group vs time

P <0.05 was adopted for statistical differences.

Abbreviations: NEFA: Non-esterified fatty acids; BHB: β-hydroxybutyrate; TP: total protein; TG: triglycerides; TC: total cholesterol; Hp: haptoglobin; BW: body weight; BCS: body condition score.

**Table S5.** Relationship between the high and low maternal groups and the inflammatory biomarkers of Holstein calves during the neonatal period (result expressed by *P*-value from the mixed linear model).

| Maternal groups | Calf biochemical parameters | | | | | | | | |
| --- | --- | --- | --- | --- | --- | --- | --- | --- | --- |
|  | Haptoglobin | | | Total Protein | | | Albumin | | |
|  | G | T | G x T | G | T | G x T | G | T | G x T |
| NEFA | 0.3780 | **0.0050** | **0.0100** | 0.0730 | **<0.001** | 0.8510 | **0.0290** | **<0.0001** | 0.8300 |
| BHB | 0.1649 | **0.0017** | 0.4363 | 0.4930 | 0.2345 | 0.3608 | 0.2779 | **<0.0001** | 0.2684 |
| Glucose | 0.2063 | **0.0009** | **0.0180** | 0.6443 | 0.2348 | 0.3689 | 0.1026 | **<0.0001** | 0.9357 |
| TP | 0.7250 | **0.0017** | 0.4879 | 0.2549 | 0.2382 | 0.4724 | 0.5679 | **<0.0001** | 0.9418 |
| Albumin | 0.3692 | **0.0017** | 0.2663 | 0.2081 | 0.2369 | 0.4309 | 0.5349 | **<0.0001** | 0.8453 |
| TG | 0.8059 | **0.0019** | 0.6149 | 0.1389 | 0.2335 | 0.3325 | 0.1887 | **<0.0001** | 0.3804 |
| TC | 0.9619 | **0.0017** | 0.7631 | 0.1843 | **<0.0001** | 0.8861 | 0.5980 | **<0.0001** | 0.5127 |
| Hp | 0.4009 | **0.0020** | 0.7502 | 0.5906 | 0.2871 | 0.1713 | 0.3679 | **<0.0001** | 0.7313 |
| Iron | 0.8943 | **0.0016** | 0.2357 | 0.5814 | **<0.0001** | 0.4249 | 0.5814 | **<0.0001** | 0.4249 |
| BW | 0.1614 | **0.0015** | 0.2252 | 0.1270 | **<0.0001** | 0.0558 | 0.0859 | **<0.0001** | **0.0060** |
| BCS | 0.5835 | 0.2533 | 0.1484 | 0.8849 | 0.4989 | 0.9349 | 0.3234 | **<0.0001** | 0.3125 |
| Season | 0.5557 | **<0.0001** | **<0.0001** | 0.9238 | 0.1977 | 0.4662 | **0.0003** | **<0.0001** | 0.0976 |

The mixed linear model was used to evaluate the main effects of group (low and high), time, and group-time interaction. G = group; T = time; G x T = group vs time

P<0.05 was adopted for statistical differences.

Abbreviations: NEFA: Non-esterified fatty acids; BHB: β-hydroxybutyrate; TP: total protein; TG: triglycerides; TC: total cholesterol; Hp: haptoglobin; BW: body weight; BCS: body condition score.

**Table S6.**  Relationship between the high and low maternal groups and the ROS production (response ratio) of cells stimulated by Phorbol Myristate Acetate (PMA), *E. coli,* *S. aureus,* and *S. hyicus* of Holstein calves during the neonatal period (result expressed by *P*-value from the mixed linear model).

| Maternal groups | ROS production (response ratio) | | | | | | | | | | | |
| --- | --- | --- | --- | --- | --- | --- | --- | --- | --- | --- | --- | --- |
|  | Cell stimulated by PMA | | | *S. aureus* pure | | | *E. coli* pure | | | *S. hyicus* pure | | |
|  | G | T | G x T | G | T | G x T | G | T | G x T | G | T | G x T |
| NEFA | 0.5826 | **0.0027** | 0.8524 | 0.3401 | **<0.0001** | 0.1145 | 0.5025 | **<0.0001** | 0.0715 | 0.0598 | **<0.0001** | 0.1007 |
| BHB | 0.2221 | **0.0023** | 0.5736 | 0.0856 | **<0.0001** | 0.2962 | 0.1917 | **<0.0001** | 0.3955 | 0.0980 | **<0.0001** | 0.6108 |
| Glucose | 0.1938 | **0.0029** | 0.7672 | 0.4768 | **<0.0001** | 0.5956 | 0.2369 | **<0.0001** | 0.4856 | 0.2969 | **<0.0001** | 0.8328 |
| TP | 0.8063 | **0.0023** | 0.4601 | 0.3963 | **<0.0001** | 0.4900 | 0.3466 | **<0.0001** | 0.3216 | 0.4065 | **<0.0001** | 0.5079 |
| Albumin | 0.6373 | **0.0013** | **0.0218** | 0.7575 | **<0.0001** | **0.0334** | 0.5462 | **<0.0001** | 0.1010 | 0.6678 | **<0.0001** | **0.0298** |
| TG | 0.8095 | **0.0024** | 0.3122 | 0.5548 | **<0.0001** | 0.5548 | 0.3200 | **<0.0001** | 0.3200 | 0.2913 | **<0.0001** | **0.0003** |
| TC | 0.9238 | **0.0030** | 0.1493 | 0.8510 | **<0.0001** | **0.0121** | 0.6849 | **<0.0001** | **0.0238** | 0.4483 | **<0.0001** | **0.0273** |
| Hp | 0.3947 | **0.0026** | 0.9106 | 0.4944 | **<0.0001** | 0.6833 | 0.7933 | **<0.0001** | 0.5580 | 0.8040 | **<0.0001** | 0.7930 |
| BW | 0.0596 | **0.0037** | 0.1601 | 0.1998 | **<0.0001** | 0.8461 | 0.3203 | **<0.001** | 0.7589 | 0.1596 | **<0.0001** | 0.8199 |
| BCS | 0.2541 | 0.1367 | 0.7504 | **0.0233** | **<0.0001** | **0.0046** | **0.0237** | **<0.0001** | 0.1153 | 0.1150 | **<0.0001** | **0.0002** |
| Season | **0.0003** | **0.0004** | **<0.0001** | **0.0004** | **<0.0001** | 0.2340 | **0.0029** | **<0.0001** | **0.0025** | **0.0008** | **<0.0001** | **0.0505** |

A mixed linear model was used to evaluate the main effects of group (low and high), time, and group-time interaction. G = group; T = time; G x T = group vs time

P <0.05 was adopted for statistical differences.

Abbreviations: NEFA: Non-esterified fatty acids; BHB: β-hydroxybutyrate; TP: total protein; TG: triglycerides; TC: total cholesterol; Hp: haptoglobin; BW: body weight; BCS: body condition score.

**Table S7.** Relationship between the high and low maternal groups and the serum IgG levels of Holstein calves during the neonatal period (result expressed by *P*-value from the mixed linear model).

| Maternal groups | Calf IgG levels | | |
| --- | --- | --- | --- |
|  | Group | Time | Group x Time |
| NEFA | 0.5210 | **<0.0001** | 0.8020 |
| BHB | 0.2727 | **<0.0001** | 0.4586 |
| Glucose | 0.3687 | **<0.0001** | 0.5319 |
| TP | 0.4057 | **<0.0001** | 0.8409 |
| Albumin | 0.0825 | **<0.0001** | 0.2223 |
| TG | 0.4197 | **<0.0001** | 0.5569 |
| TC | 0.3084 | **<0.0001** | 0.4070 |
| Hp | 0.6566 | **<0.0001** | 0.3599 |
| BW | 0.1790 | **<0.0001** | 0.2532 |
| BCS | 0.5667 | **<0.0001** | 0.4024 |
| Season | **0.0312** | **<0.0001** | 0.2528 |

A mixed linear model was used to evaluate the main effects of group (low and high), time, and group-time interaction. G = group; T = time; G x T = group vs time

P <0.05 was adopted for statistical differences.

Abbreviations: NEFA: Non-esterified fatty acids; BHB: β-hydroxybutyrate; TP: total protein; TG: triglycerides; TC: total cholesterol; Hp: haptoglobin; BW: body weight; BCS: body condition score.

**Table S8.** Diarrhea frequency (absolute number/total sample size - %) of Holstein calves during neonatal period divided by the different maternal groups.

| Maternal groups | D1 | | D2 | | D7 | | D14 | | D28 | |
| --- | --- | --- | --- | --- | --- | --- | --- | --- | --- | --- |
|  | Low | High | Low | High | Low | High | Low | High | Low | High |
| NEFA | 7.14%  (1/14) | 0.00%  (0/14) | 21.42%  (3/14) | 7.14% (1/14) | 14.28%  (2/14) | 42.85%  (6/14) | 42.85%  (6/14) | 28.57%  (4/14) | 28.57%  (4/14) | 35.71%  (5/14) |
| BHB | 7.69%  (1/13) | 0.00% (0/15) | 15.38%  (2/13) | 13.33%  2/15) | 46.15%  (6/13) | 13.33%  (2/15) | 23.07%  (3/13) | 40.00%  (6/15) | 46.15%  (6/13) | 20.00%  (3/15) |
| Glucose | 0.00%  (0/14) | 7.14%  (1/14) | 14.28%  (2/14) | 14.28%  (2/14) | 35.71%  (5/14) | 21.42%  (3/14) | 21.42%  (3/14) | 50.00%  (7/14) | 42.85%  (6/14) | 21.42%  (3/14) |
| TP | 0.00%  (0/14) | 7.14% (1/14) | 14.28%  (2/14) | 14.28%  (2/14) | 42.85%  (6/14) | 14.28%  (2/14) | 21.42%  (3/14) | 50.00%  (7/14) | 35.71%  (5/14) | 28.57%  (4/14) |
| Albumin | 0.00%  (0/14) | 7.14% (1/14) | 14.28%  (2/14) | 14.28%  (2/14) | 35.71%  (5/14) | 21.42%  (3/14) | **14.28% A**  **(2/14)** | **57.14% B**  **(8/14)** | 28.57%  (4/14) | 35.71%  (5/14) |
| TG | 0.00%  (0/14) | 7.14% (1/14) | 7.14% (1/14) | 21.42%  (3/14) | 28.57%  (4/14) | 28.57%  (4/14) | 21.42%  (3/14) | 50.00%  (7/14) | 28.57  (4/14) | 35.71%  (5/14) |
| TC | 0.00%  (0/13) | 6.67%  (1/15) | 7.69%  (1/13) | 20.00%  (3/15) | 30.76%  (4/13) | 26.66%  (4/15) | 23.07%  (3/13) | 46.66%  (7/15) | 23.07%  (3/13) | 40.00%  (6/15) |
| Hp | 0.00%  (0/13) | 6.67%  (1/15) | 23.07%  (3/13) | 6.66%  (1/15) | 30.76%  (4/13) | 26.66%  (4/15) | 46.15%  (6/13) | 26.66%  (4/15) | 38.46%  (5/13) | 26.66%  (4/15) |
| BW | 0.00%  (0/14) | 7.14% (1/14) | 14.28%  (2/14) | 14.28%  (2/14) | 28.57%  (4/14) | 28.57%  (4/14) | 28.57  (4/14) | 42.85%  (6/14) | 42.85%  (6/14) | 21.42%  (3/14) |
| BCS | 0.00%  (0/14) | 7.14% (1/14) | 14.28%  (2/14) | 14.28%  (2/14) | 14.28%  (2/14) | 42.85%  (6/14) | 42.85%  (6/14) | 28.57%  (4/14) | **14.28% A**  **(2/14)** | **50.00% B**  **(7/14)** |
| Season | 0.00%  (0/14) | 7.14% (1/14) | 7.14% (1/14) | 21.42%  (3/14) | 21.42%  (3/14) | 35.71%  (5/14) | 42.85%  (6/14) | 28.57%  (4/14) | **0.00% A**  **(0/14)** | **64.28% B**  **(9/14)** |

A, B: different letters on the same line indicate statistical differences between groups in comparison with the chi-square test (P <0.05).

Abbreviations: NEFA: Non-esterified fatty acids; BHB: β-hydroxybutyrate; TP: total protein; TG: triglycerides; TC: total cholesterol; Hp: haptoglobin; BW: body weight; BCS: body condition score.

**Table S9.** Frequency (absolute number/total sample size - %) of bovine respiratory disease (BRD) score ≥ 4 of Holstein calves during the neonatal period divided by the different maternal groups.

| Maternal groups | D1 | | D2 | | D7 | | D14 | | D28 | |
| --- | --- | --- | --- | --- | --- | --- | --- | --- | --- | --- |
|  | Low | High | Low | High | Low | High | Low | High | Low | High |
| NEFA | 7.14%  (1/14) | 7.14%  (1/14) | 0.00%  (0/14) | 7.14% (1/14) | 0.00%  (0/14) | 21.42%  (3/14) | 14.28%  (2/14) | 28.57%  (4/14) | 28.57%  (4/14) | 28.57%  (4/14) |
| BHB | 0.00% (0/13) | 6.67% (1/15) | 7.69%  (1/13) | 0.00%  (0/15) | 0.00%  (0/13) | 20.00%  (3/15) | 30.76%  (4/13) | 13.33%  (2/15) | 30.76%  (4/13) | 26.66%  (4/15) |
| Glucose | 0.00%  (0/14) | 7.14%  (1/14) | 0.00%  (0/14) | 7.14%  (1/14) | 7.14%  (1/14) | 14.28%  (2/14) | 35.71%  (5/14) | 7.14%  (1/14) | 28.57%  (4/14) | 28.57%  (4/14) |
| TP | 0.00%  (0/14) | 7.14% (1/14) | 0.00%  (0/14) | 7.14%  (1/14) | 14.28%  (2/14) | 7.14%  (1/14) | 21.42%  (3/14) | 21.42%  (3/14) | 35.71%  (5/14) | 21.42%  (3/14) |
| Albumin | 14.28%  (2/14) | 0.00% (0/14) | 0.00%  (0/14) | 7.14%  (1/14) | 14.28%  (2/14) | 7.14%  (1/14) | 7.14%  (1/14) | 35.71%  (5/14) | 21.42%  (3/14) | 35.71%  (5/14) |
| TG | 14.28%  (2/14) | 0.00% (0/14) | 0.00%  (0/14) | 7.14%  (1/14) | 14.28%  (2/14) | 7.14%  (1/14) | 7.14%  (1/14) | 35.71%  (5/14) | 21.42%  (3/14) | 35.71%  (5/14) |
| TC | 15.38%  (2/13) | 0.00%  (0/15) | 0.00%  (0/13) | 6.67%  (1/15) | 23.07%  (3/13) | 0.00%  (0/15) | 15.38%  (2/13) | 26.66%  (4/15) | 23.07%  (3/13) | 33.33%  (5/15) |
| Hp | 0.00%  (0/13) | 6.67%  (1/15) | 7.69%  (1/13) | 0.00%  (0/15) | 0.00%  (0/13) | 20.00%  (3/15) | 38.46%  (5/13) | 6.67%  (1/15) | 30.76%  (4/13) | 26.66%  (4/15) |
| Iron | 0.00%  (0/14) | 7.14% (1/14) | 7.14%  (1/14) | 0.00%  (0/14) | 7.14%  (1/14) | 14.28%  (2/14) | 35.71  (5/14) | 7.14%  (1/14) | 42.85%  (6/14) | 14.28%  (2/14) |
| BW | 7.14%  (1/14) | 7.14% (1/14) | 0.00%  (0/14) | 7.14%  (1/14) | 7.14%  (1/14) | 14.28%  (2/14) | 14.28%  (2/14) | 28.57%  (4/14) | 35.71%  (5/14) | 21.42%  (3/14) |
| BCS | 14.28%  (2/14) | 0.00% (0/14) | 0.00%  (0/14) | 7.14%  (1/14) | 21.42%  (3/14) | 0.00%  (0/14) | 35.71%  (5/14) | 7.14%  (1/14) | 42.85  (6/14) | 14.28%  (2/14) |

No differences were found between maternal groups in comparison with the chi-square test (P>0.05).

Abbreviations: NEFA: Non-esterified fatty acids; BHB: β-hydroxybutyrate; TP: total protein; TG: triglycerides; TC: total cholesterol; Hp: haptoglobin; BW: body weight; BCS: body condition score.
